# Collaborative Care in NSCLC; the Role of Early Palliative Care

**DOI:** 10.3389/fonc.2014.00192

**Published:** 2014-07-30

**Authors:** Marnie Howe, Ronald L. Burkes

**Affiliations:** ^1^Department of Family and Community Medicine, Division of Palliative Medicine, University of Toronto, Toronto, ON, Canada; ^2^Department of Medicine, Division of Medical Oncology, University of Toronto, Toronto, ON, Canada

**Keywords:** collaborative care, NSCLC, early palliative care

## Abstract

The management of non-small cell lung cancer (NSCLC) has evolved into a multidisciplinary team approach that traditionally has involved medical oncology, radiation oncology, and thoracic surgery. However, in the era of personalized medicine the importance of molecular diagnostics requires adequate tissue for histologic subtyping and molecular testing and thus requires the engagement of other subspecialties such as pathology, respirology, and interventional radiology. Unfortunately in 2014, the majority of patients presenting with NSCLC will succumb to their disease and the early integration of palliative care into the treatment strategy will improve the quality of life and end-of-life care of our patients and may in fact improve their overall survival.

## Introduction

Lung cancer is globally the leading cause of cancer death for both men and women, more so than colorectal, breast and prostate cancer combined (1). According to the Canadian Cancer Society’s statistics 2013 report, lung cancer accounts for approximately 25% of cancer deaths each year (2). Histologically, the majority of lung cancers diagnosed are non-small cell lung cancer (NSCLC). The most important risk factor for developing lung cancer remains tobacco use, accounting for an estimated 86% of lung cancer cases in high-income countries like Canada and the median age at diagnosis is 70. However, the incidence of lung cancer in non-smoking young women is increasing (2).

Although there is no data available specifically on NSCLC in Canada, the National Cancer Institute’s surveillance, epidemiology, and end results (SEER) database in the United States has reported 5-year survival rates across all stages being only 16%, with stage IIIA, IIIB, and IV at 14, 5, and 1%, respectively (3). Although post-operative adjuvant treatment, combined-modality treatment approaches and the newer targeted therapies have improved outcomes in a subset of patients, NSCLC continues to be diagnosed late with the majority of patients presenting with advanced stage IV disease with an associated significant symptom burden. Thus, the goal of systemic therapy in this setting is to prolong survival and improve quality of life (QoL) by treating cancer related symptoms.

Dr. Ellis has previously written an excellent review of the importance of the multidisciplinary team management of patients with NSCLC (4). In this review, he addressed such things as diagnostic assessment clinics, multidisciplinary case conferencing, and involvement of the patient as part of the team as well as psychosocial and nutritional support. Thus, the primary focus of this review will be on the early integration of palliative care. As well we will briefly address the rationale for an increased awareness of recent advances in lung cancer care among the various specialties involved in the assessment of patients with lung cancer and the need to establish a cohesive network for ongoing communication and collaboration.

## One Example of the Importance of a Collaborative Care Approach to Non-Small Cell Lung Cancer

Although chemotherapy remains the standard of care for most patients, there has clearly been a shift toward personalized therapy with the understanding of molecular diagnosis and treatment of lung cancer. This has come about by the identification of a number of actionable mutations including the EGFR and ALK fusion genes, which have revolutionized therapy in those patients who harbor these mutations (5–7). As demonstrated by the Lung Cancer Mutation Consortium, two-thirds of NSCLC patients have an oncogenic driver and those patients with these drivers have been shown to live longer if they receive the corresponding targeted agent (8).

Unfortunately, the majority of patients present to the medical oncologist without knowledge of the EGFR or ALK mutation status, which has the potential for delaying the start of treatment. Furthermore, a significant number of patients will be found to have insufficient tissue to do the appropriate molecular analysis, which will further delay treatment and often requires commencing chemotherapy in patients in whom it might be unnecessary thus exposing them to increased toxicity and an inferior treatment. Therefore, it is important for physicians involved in the diagnosis and treatment of lung cancer to be aware that maximizing tissue yield for histologic subtyping and molecular testing is not only crucial but also essential to be able to offer patients a personalized treatment approach.

Thus, an increased awareness of recent advances in lung cancer care by engaging multidisciplinary teams in discussions about innovative knowledge transfer strategies would hopefully lead to more effective practice. It is also important for professionals including respirologists, thoracic surgeons, internal medicine, and interventional radiologists working with lung cancer patients to establish a cohesive network for ongoing communication and collaboration.

## Integration of Early Palliative Care

A study examining inoperable NSCLC patients with a good performance status found an average of 14.3 symptoms reported by these patients at presentation. Even patients with a WHO Performance Status of 0 (fully active) reported an average of 11.6 symptoms. The most commonly reported symptoms included fatigue, lack of energy, shortness of breath, cough, worrying, and chest pain (9). Similarly, a Canadian study conducted in the ambulatory setting found newly diagnosed lung cancer patients had a greater symptom burden compared to other cancer sites (10). As expected, as lung cancer progresses, so does the symptom burden. A group of patients in a community radiation oncology program were found to have increased symptom severity during the last few months of their life, compared to the 3-months prior (11). These studies highlight the importance of early palliative care (EPC) involvement to help guide symptom management.

Palliative care has also been shown to assist with goals of care discussions and address psychosocial supports. The WHO now defines palliative care as “an approach that improves the quality of life of patients and their families facing the problem associated with life-threatening illness, through the prevention and relief of suffering by means of early identification and impeccable assessment and treatment of pain and other problems including physical, psychosocial, and spiritual.” As well they state “is applicable early in the course of illness, in conjunction with other therapies that are intended to prolong life, such as chemotherapy or radiation therapy, and includes those investigations needed to better understand and manage distressing clinical complications” (12). Patients report inadequate communication with providers about shared decision-making at the end-of-life (EOL). This better understanding of their illness through important conversations can help patients and families prepare for EOL, which they have reported as a valuable aspect of their care. In fact, patients rely on their physicians to discuss hospice, advanced directives, and other EOL care options. Studies have shown deficiencies in the area of communication surrounding EOL (13, 14) and palliative care can assist in facilitating these important discussions.

The American Society of Clinical Oncology published a provisional clinical opinion in 2012 recommending the integration of palliative care into standard oncology care for patients with metastatic cancer. This recommendation was based on a phase III clinical trial conducted by Temel and colleagues of 151 ambulatory patients newly diagnosed with NSCLC. Patients were randomly assigned to standard oncology care or EPC. The primary outcome was QoL at 12 weeks, with secondary outcomes including mood, understanding of illness, and aggressiveness of care at the EOL. Both QoL and depression had significantly improved with EPC. In addition, patients in the intervention group had higher documentation rates of resuscitation preferences, as well as less aggressive care at EOL, including intravenous chemotherapy in the last 2 weeks of life. Despite this, patients who received EPC had a significantly prolonged survival, by approximately 3 months (15).

A recent review by Irwin et al. postulated the possible mechanisms for prolonged survival attributed to EPC in patients with metastatic NSCLC. They identified four randomized controlled trials, including the Temel study above, which studied palliative care interventions’ effects on QoL and EOL care in cancer patients. They all found improved QoL in patients who received EPC, and two of the studies showed increased survival in the intervention groups. Irwin and colleagues hypothesized several variables are associated with increased survival in this population based on current literature (16). The first being palliative care’s focus on relieving suffering and improving symptom distress. Both health-related QoL and physical symptoms have been associated with prognosis (17, 18). Secondly, they identified treating depression as a potential mechanism for improved survival. The relationship between depression and survival may be due to multiple effects. Improving depression may have a beneficial impact on health behaviors linked to treatment adherence and well-being. Depression is known to activate the hypothalamic–pituitary axis and thus increase levels of cortisol, affecting the immune system and specifically, helper T cells (19, 20). A third mechanism identified is EPC’s focus on disease/prognosis understanding and goals of care discussions. When patients had a better understanding of their prognosis, they were less likely to receive chemotherapy at EOL. Many believe that there is a point at EOL where the toxicity of chemotherapy may hasten death. Temel et al. found patients assigned to EPC were less likely to receive intravenous chemotherapy at EOL and the interval between last chemotherapy infusion and time of death was longer for patients who received EPC compared to those who did not (16). Finally, the focus on increasing social support for both the patient and their caregivers may also be a factor in extending length of survival. Many studies have demonstrated that marital status is associated with a survival advantage for lung cancer patients, which may translate into increased social support (21, 22).

In a more recent retrospective study, Hui and colleagues (23) looked at the impact, timing, and setting of palliative care referrals on the quality of end-of-life in cancer patients. A total of 366 adult cancer patients at a tertiary care center who between September 1, 2009 and February 28, 2010 who received a palliative care referral and who had contact with the cancer center within the last 3 months of life were included in the study. Outpatient referrals were associated with significantly fewer emergency room visits, hospital admissions, hospital deaths, ICU admissions, and a shorter duration of hospital stay, when compared to inpatient referrals. Outpatient palliative care referral remained an independent factor for improved end-of-life care in multivariate analysis. It is noteworthy that 20% of patients in each group had a diagnosis of lung cancer. Early outpatient involvement with palliative care allows patients to develop a longitudinal therapeutic relationship. Through the course of multiple clinic visits the palliative care team can help facilitate goals of care discussions and advanced care planning, with the hope of reducing aggressive interventions at EOL. The palliative care team also has access to community resources and supports for both the patient and their families as their disease progresses. Finally, the palliative care focus on symptom assessments not only improves QoL but can also minimize unnecessary ER or hospital admissions with patient education and routine follow-up for symptom management (23).

## Conclusion

Clearly, our ultimate goal is to improve the diagnosis and care of lung cancer patients. The treatment of NSCLC has evolved considerably including the introduction of post-operative adjuvant therapy for early stage disease and the use of combined-modality therapy for stage III disease that requires the collaboration of medical oncology, radiation oncology, and thoracic surgery. The management of advanced disease has also evolved significantly and a personalized approach to treatment in advanced stage IV disease is a reality. Although traditionally, collaboration has primarily been between thoracic surgery, radiation oncology, and medical oncology, in the era of personalized medicine it is important for all physicians involved in the diagnosis and treatment of lung cancer to be aware that maximizing tissue yield for histologic subtyping and molecular testing is not only crucial but also essential to be able to offer patients the appropriate therapy in a timely fashion. Unfortunately in 2014, the majority of patients presenting with NSCLC will succumb to their disease and studies have shown that the early integration of palliative care into the management strategy will improve the QoL and EOL care of our patients.

## Conflict of Interest Statement

The authors declare that the research was conducted in the absence of any commercial or financial relationships that could be construed as a potential conflict of interest.
